# Genetic Basis and Expression Pattern Indicate the Biocontrol Potential and Soil Adaption of *Lysobacter capsici* CK09

**DOI:** 10.3390/microorganisms11071768

**Published:** 2023-07-06

**Authors:** Pu Yang, Chaofan Qu, Miaomiao Yuan, Bo Xi, Xiu Jia, Ben Zhang, Lizhen Zhang

**Affiliations:** 1 School of Life Science, Shanxi University, Taiyuan 030006, China; yangpu@sxu.edu.cn (P.Y.); 17636269588@163.com (C.Q.); 18835398412@163.com (M.Y.); 19581557939@163.com (B.X.); benzhang@sxu.edu.cn (B.Z.); 2 Institute of Biodiversity, Faculty of Biological Sciences, Cluster of Excellence Balance of the Microverse, Friedrich Schiller University Jena, 07745 Jena, Germany; xiu.jia@uni-jena.de

**Keywords:** *Lysobacter capsici*, biocontrol, lytic enzymes, secretome, adaption

## Abstract

*Lysobacter* species have attracted increasing attention in recent years due to their capacities to produce diverse secondary metabolites against phytopathogens. In this research, we analyzed the genomic and transcriptomic patterns of *Lysobacter capsici* CK09. Our data showed that *L. capsici* CK09 harbored various contact-independent biocontrol traits, such as fungal cell wall lytic enzymes and HSAF/WAP-8294A2 biosynthesis, as well as several contact-dependent machineries, including type 2/4/6 secretion systems. Additionally, a variety of hydrolytic enzymes, particularly extracellular enzymes, were found in the *L. capsici* CK09 genome and predicted to improve its adaption in soil. Furthermore, several systems, including type 4 pili, type 3 secretion system and polysaccharide biosynthesis, can provide a selective advantage to *L. capsici* CK09, enabling the species to live on the surface in soil. The expression of these genes was then confirmed via transcriptomic analysis, indicating the activities of these genes. Collectively, our research provides a comprehensive understanding of the biocontrol potential and soil adaption of *L. capsici* CK09 and implies the potential of this strain for application in the future.

## 1. Introduction

Crops are often threatened by phytopathogens, such as *Fusarium*, *Pythium* and *Alternaria* [1,2,3]. These pathogens induce necrosis and cell death via virulence factors, and hence, cause plant diseases and yield losses [3,4,5]. In agricultural application, several emerging strategies, such as suppressive soil and biocontrol microorganisms, have been exploited in pathogen control [6,7]. These biocontrol microorganisms, including *Bacillus*, *Lysobacter*, *Pseudomonas* and *Trichoderma*, are regarded as environmentally friendly agents, and combat phytopathogens via competition, mycoparasitism, antibiosis and the activation of host defense system [8,9,10].

*Lysobacter* species are one group of these biocontrol microorganisms that are widely distributed in suppressive soil [11,12]. The higher abundance of *Lysobacter* species in the rhizosphere is related to a stronger suppression effect on plant pathogens [13]. Several species, such as *Lysobacter capsici* [14], *Lysobacter antibioticus* [15,16] and *Lysobacter enzymogenes* [17], have been applied to fight against plant fungal pathogens [14,17], bacterial pathogens [15] and worms [16]. *Lysobacter* species utilize various antimicrobial mechanisms. They produce a variety of lytic enzymes, including lysozyme [18], chitinase [19], β-glucanase [20] and protease/peptidase [21,22], and thus degrade the cell walls of fungi and bacteria. Additionally, they produce various secondary metabolites, such as phenazine [15], heat-stable antifungal factor (HSAF) [23], hypeptine [24], lysocin E [25] and 2,5-diketopiperazines [26], which were identified as antimicrobial agents. Volatile compounds produced by *Lysobacter* species also contribute to their antimicrobial activity [27], while certain *Lysobacter* species may induce systemic resistance in plants [28]. Thus, *Lysobacter* species show potential as biocontrol agents in agriculture.

Adaption to and survival in soil and the rhizosphere are key factors for biocontrol efficiency. The survival of bacterial cells in soil is influenced by various soil characteristics, such as the indigenous bacterial and fungal community, temperature, soil type, pH, electronic conductivity and nitrate [29,30]. Bacterial traits, such as polyphosphate kinase [31], type three secretion system [32], pilus- and flagella-mediated motility [33] and biofilm formation [34], are also related to bacterial adaption in soil.

In a previous study, a strain with excellent biocontrol activities, *Lysobacter capsici* CK09, was isolated from the rhizosphere of *Caragana Korshinskii* Kom [35]. *L. capsici* CK09 showed antimicrobial activities against several Gram-positive and Gram-negative bacterial pathogens, as well as fungal phytopathogens [35]. However, the ways in which *L. capsici* CK09 strains inhibit microbial growth and adapt to soil environments remain unknown. In this research, we aimed to elucidate the mechanisms involved in the biocontrol ability and soil adaption of *L. capsici* CK09 through complete genomic sequencing and transcriptomic analysis.

## 2. Material and Methods

### 2.1. Genome Sequencing, Assembly and Annotation

*L. capsici* CK09, which was isolated by Ma [35], was cultured in R2A broth with shaking at 28 °C. The bacterial cells of the 24 h culture were collected via centrifugation and applied to genomic DNA extraction, which was performed using the QIAGEN genomic DNA extraction kit (QIAGEN, Hilden, Germany), following the manufacturer’s instruction. Then, the purified DNA was sequenced using the MGISEQ-2000 platform (MGI Tech Co., Ltd., Shenzhen, China) and the PromethION sequencer (Oxford Nanopore Technology, ONT, Oxford, UK) with the assistance of Shanghai Biotree Biotech (Shanghai, China), according to the manufacturer’s instruction. To construct the library for ONT sequencing, the PippinHT system (Sage Science Inc., Beverly, MA, USA) was used for recovering the long DNA, the NEBNext Ultra II End Repair/dA-tailing Kit (New England Biolabs Inc., Ipswich, MA, USA) was used in A-ligation and the SQK-LSK109 kit (Oxford Nanopore Technology) was used for adapter ligation. For the MGISEQ sequencing, 200–400 bp of fragmented DNA was purified using the Agencourt AMPure XP kit (Beckman Coulter Inc., Pasadena, CA, USA). The produced sequences, with a mean q-score template ≥7 and length ≥1000 bp from ONT were selected for genome assembly using Flye 2.6 (https://github.com/fenderglass/Flye, accessed on 10 June 2020). Then, the assembled ONT sequencing data were corrected with Pilon 1.23 (https://github.com/broadinstitute/pilon, accessed on 12 June 2020) and Nextpolish 1.4.7 (https://github.com/Nextomics/NextPolish, accessed on 12 June 2020) based on the DNA sequence generated with MGISEQ-2000. Finally, the replication origin was set as base 0 using Circlator 1.5.1 (https://github.com/sanger-pathogens/circlator, accessed on 12 June 2020).

The coding sequences were predicted using Prodial 2.6.3 (https://github.com/hyattpd/Prodigal, accessed on 14 June 2020), tRNA genes were analyzed using tRNAscan-SE 2.0 (http://lowelab.ucsc.edu/tRNAscan-SE, accessed on 14 June 2020) [36], rRNAs were determined using RNAmmer 1.2 (http://www.cbs.dtu.dk/services/RNAmmer, accessed on 14 June 2020) [37] while other non-coding RNAs were assessed in Infernal by searching the Rfam database (http://rfam.xfam.org/, accessed on 14 June 2020) [38].

Functional annotation of the proteins was performed via searching the TIGRFAMs, Pfam and GO, KEGG, Refseq and COG databases. The average nucleotide identity (ANI) was calculated using JSpeciesWS (http://jspecies.ribohost.com/jspeciesws/, accessed on 2 April 2022) [39]. For ANI analysis, the genomic sequences of 22 *Lysobacter* strains were downloaded from NCBI. The secondary metabolite biosynthesis gene cluster analysis was conducted on antiSMASH (https://antismash.secondarymetabolites.org/#!/start, accessed on 20 April 2022) [40]. The signal peptides were examined via SignalIP 6.0 (https://services.healthtech.dtu.dk/service.php?SignalP-6.0, accessed on 1 May 2022) [41]. The predicted proteins were manually reviewed using BLASTP against the verified proteins in the previous reports or using the Pfam database (https://pfam.xfam.org/, accessed on 15 June 2020) to determine the conserved domains.

### 2.2. Transcriptomic Sequencing and Annotation

The 24 h culture of *L. capsici* CK09 was inoculated in R2A broth with shaking at 28 °C. The initial cell density was 10^7^ CFU/mL, which was determined using the plate count method immediately after inoculation. The cells for RNA extraction were collected with centrifugation at 24 h and 72 h after inoculation.

RNA extraction was performed using TRIzol reagent (Invitrogen, Thermo Fisher Scientific Inc., Waltham, MA, USA) according to the manufacturer’s instructions. Then, genomic DNA was omitted with DNaseI (Takara Bio Inc., Kusatsu-shi, Japan) before RNA-seq transcriptome library construction, which was performed using the TruSeq RNA sample prep kit (Illumina Inc., San Diego, CA, USA). In this procedure, the Ribo-Zero Magnetic kit (Epicenter, Illumina Inc.) was utilized to remove the rRNA. The resulting library was sequenced with Illumina HiSeq × TEN at Shanghai Majorbio Bio-pharm Biotechnology Co., Ltd. (Shanghai, China). The data were analyzed via the online Major Cloud Platform (www.majorbio.com, accessed on 14 November 2022) [42] based on the genome information given above.

Three biological replicates were used in the transcriptomic analysis. Differentially expressed genes were selected using the criterion with log_2_FC (fold change) >1 or <−1, and *p* value < 0.05.

## 3. Results

### 3.1. General Features of the L. capsici CK09 Genome and Transcriptomic Data

The chromosome size of *L. capsici* CK09 was 6,215,653 bp, while the G + C content was 66.78%. The genome of *L. capsici* CK09 consisted of 4995 predicted protein-coding sequences, corresponding to 83.64% of the genome. Additionally, 52 tRNAs, 6 rRNAs and 27 other non-coding rRNAs were present in the assembled genome. No plasmid was found in *L. capsici* CK09.

**Figure 1:**
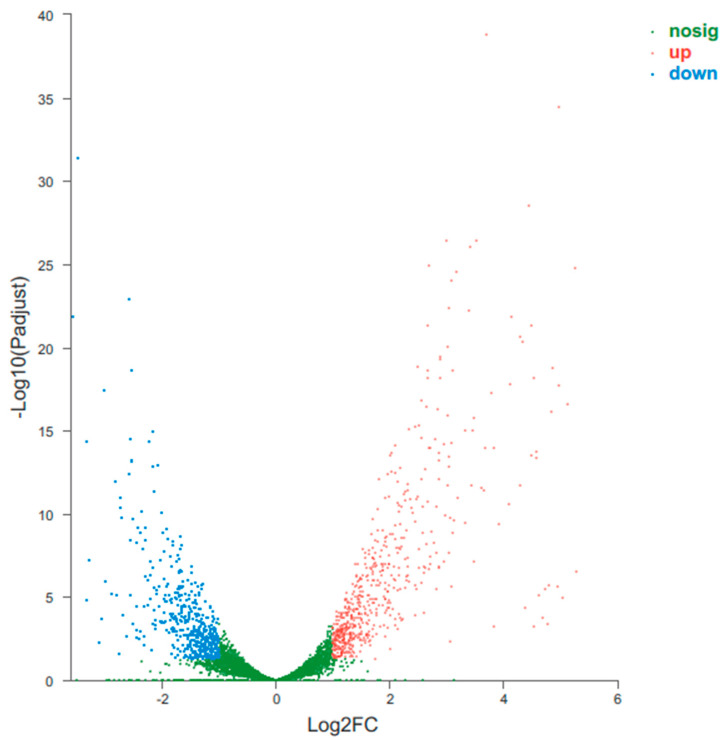
Expression pattern of *L. capsici* CK09 between the 24 h and 72 h culture.

After 24 and 72 h of inoculation, the expression of 4954 and 4947 genes were detected, comprising 99.18% and 99.04% of all the predicted genes, respectively. Among them, 4946 genes were expressed at both sampling times. Additionally, 599 genes were significantly up-regulated and 474 genes were significantly down-regulated in the 72 h culture, as compared to the 24 h culture (Figure 1).

### 3.2. ANI Analysis

**Figure 2:**
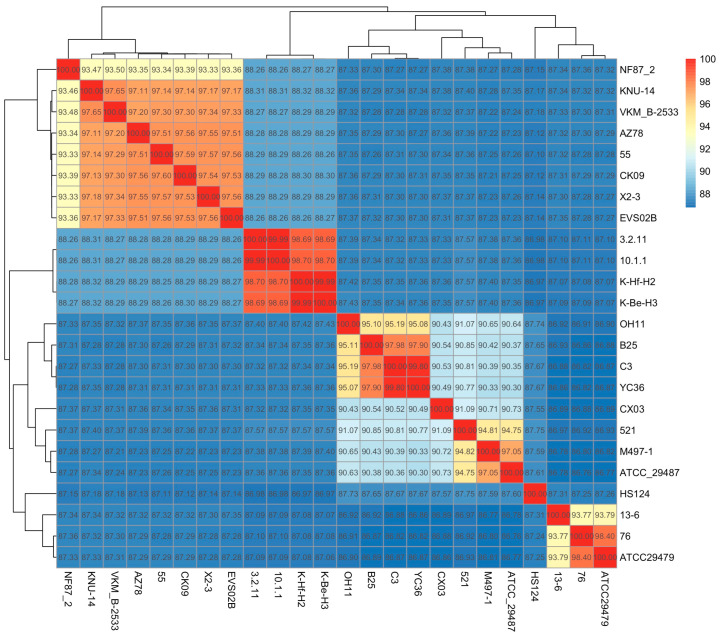
ANI matrices of *Lysobacter* strains.

The ANI is widely used in whole-genome phylogenetic analysis [43]. An ANI value of 95% was considered as the threshold for species divergence [43,44]. In this study, 23 genomes, including *L. capsici*, *L. antibioticus*, *L. enzymogenes* and *L. gummosus*, were included in an all-in-all pairwise ANI analysis. The results indicated that these *Lysobcter* species were clustered in 11 lineages (Figure 2). As shown in Figure 2, *L. capsici* CK09 was phylogenetically related to *L. capsici* KNU-14, VKM_B-2533, AZ78, 55 and X2-3, while the first was used as the reference genome in NCBI. These data confirmed the classification of *L. capsici* CK09 provided in a previous study [35]. Additionally, the strain EVS02B, which was clustered in *L. gummosus*, shared high ANI values (>95%) with *L. capsici* strains, indicating that EVS02B belonged to *L. capsici*. On the other hand, the strains K-Hf-H2 and NF87_2, which were assigned as *L. capsici*, revealed ANI values lower than 95% with the other *L. capsici* strains. The ANI values between K-Hf-H2 and *L. gummosus* K-Be-H3, 3.2.11 and 10.1.1 were greater than 95%. Thus, strain K-Hf-H2 should be regarded as *L. gummosus*. As for NF87_2, it showed ANI values lower than 95% with all the other strains in this study, suggesting a novel species in the *Lysobacter* genus. This was also the case for the strains CX03, 13-6, HS124 and 521; hence, the classification of the *Lysobacter* genus should be investigated further in the future.

### 3.3. Lytic Enzymes

*Lysobacter* can produce a variety of lytic enzymes [45]. The activities of chitinase and glucanase were verified on an agar medium [35]. Then, the coding genes of these enzymes were searched based on the protein sequences verified in previously published papers (Table S1). In addition to two chitinase-coding genes (CK09SX_10745, CK09SX_19275), *L. capsici* CK09 also carried three β-glucanase-associated genes (CK09SX_02815, CK09SX_07780, CK09SX_17895). All the genes were expressed at both 24 h and 72 h with TPMs 15.35~254.22. Among them, CK09SX_19275 and CK09SX_17895 showed significantly higher expression levels at 24 h compared to that at 72 h, while the expression levels of the other three genes were similar between the 24 h and 72 h culture (Table S1).

The protein sequences of *L. capsici* CK09 were also submitted to the dbCAN meta-server to search carbohydrate-active enzymes. The results revealed that a total of 123 proteins contained carbohydrate-active domains, including 11 auxiliary activities, 9 carbohydrate-binding modules, 16 carbohydrate esterases, 49 glycoside hydrolases, 40 glycosyl transferases and 5 polysaccharide lyases (Table S2). Five of these had two distinct domains while CK09SX_06550 contained two glycosyl transferase domains and CK09SX_11710 harbored two carbohydrate-binding modules. Meanwhile, 54 of them contained signal peptides, indicating that these proteins were extracellular enzymes. The transcriptomic data verified the expression of these genes with only two exceptions, CK09SX_02630 and CK09SX_14170.

### 3.4. Secondary Metabolite Biosynthesis and Non-Ribosomal Peptide Synthases (NRPS)

Twelve regions of secondary metabolite biosynthesis clusters were discovered in *L. capsici* CK09 genome using the antiSMASH web server (Table S3). However, nine out of these twelve regions only contained an orphan gene associated with secondary metabolite biosynthesis or secretion. The predicted protein sequences in these regions were compared to the certain proteins in the reference strains. The results suggested that these orphan proteins were not sufficient to synthesize the certain compounds. On the other hand, Region 2, Region 8 and Region 10 harbored the essential genes for the biosynthesis of the corresponding compounds.

**Figure 3:**
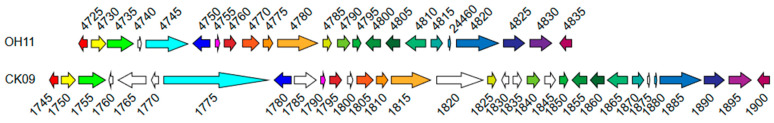
Predicted gene cluster of yellow pigment biosynthesis. Genes of the same color are homologous genes.

The colony of *L. capsici* CK09 appeared yellow. The gene cluster associated with the yellow pigment was found in the genome of *L. capsici* CK09. A total of 32 genes (Region 2, CK09SX_01745 to CK09SX_01900, Table S4) were located in the cluster, while 22 of them were identical to the related genes in *L*. *enzymogenes* OH11 [46] with several genes inserted (Figure 3). The TPMs of most genes were less than 20, whereas the TPMs of CK09SX_01875 and CK09SX_01880 were higher than 1000. However, the functions of the these two genes remain unknown.

**Figure 4:**
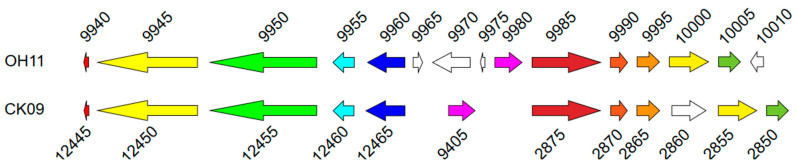
Predicted gene cluster of WAP-8294A2 biosynthesis. Genes of the same color are homologous genes.

Region 8 (CK09SX_12445 to CK09SX_12465) was predicted to be responsible for the biosynthesis of an antibacterial cyclic lipodepsipeptide, WAP-8294A2 (Table S5 and Figure 4). These proteins were identical to the proteins of the MbtH family protein (CK09SX_12445), NRPSs (CK09SX_12450 and CK09SX_12455), TauD/TfdA family dioxygenase (CK09SX_12460) and ABC transporter (CK09SX_12465) in *L. enzymogenes* OH11 [47]. However, these genes were insufficient for WAP-8294A2 biosynthesis. Thus, the protein of *L. capsici* CK09 was searched via BLASTP using the protein sequences of WAP-8294A2 biosynthesis genes from *L. enzymogenes* OH11 as the query. Another gene cluster from CK09SX_02850 to CK09SX_02875 was then discovered. Thus, *L. capsici* CK09 possessed all the genes required for WAP-8294A2 biosynthesis. All the genes were expressed similarly at the two sampling times with TPMs at 1~586.

**Figure 5:**
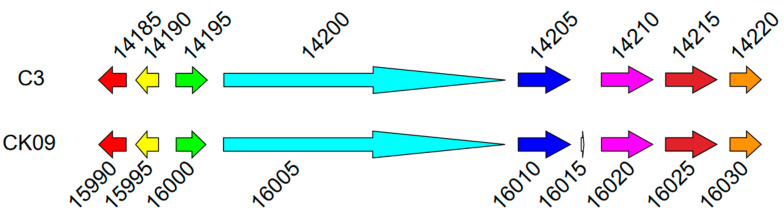
Predicted gene cluster of HSAF biosynthesis. Genes of the same color are homologous genes.

In Region 10, the gene cluster from CK09SX_15990 to CK09SX_16030 represented a complete set of genes for HSAF biosynthesis (Table S6 and Figure 5), which were reported previously in *L. enzymogenes* C3 [48]. Among them, CK09SX_15990 encoded an arginase, CK09SX_15995 generated a ferredoxin-NADP reductase, CK09SX_16000 produced a sterol desaturase family protein and CK09SX_16005 was related to an NRPS. The proteins encoded by CK09SX_16010, CK09SX_16020, CK09SX_16025 and CK09SX_16030 were NAD(P)/FAD-dependent oxidoreductase, but they played distinct roles in HSAF biosynthesis. Ferredoxin-NADP reductase and arginase were not necessary for HSAF biosynthesis, while the rest of the genes were essential. The TPMs of these were 99.28~1028.67 at 24 h and 21.25~189.55 at 72 h. Eight out the nine genes in this cluster were expressed significantly lower at 72 h culture (Table S6).

Additionally, another 10 genes were assigned as NRPS genes (Table S7). The protein products of these genes ranged from 1099 to 5689 amino acids, including three to twenty functional domains. Adenylation domain, thiolation domain and condensation domain were shared among all the NRPSs; while CK09SX_02175 also contained a thioesterase domain, CK09SX_02300 had a reduction domain; CK09SX_12515 and CK09SX_12545 possessed methyltransferase domains; and CK09SX_12530 harbored ketoacyl-synthetase and acyl transferase domains. The expression of these genes was verified (Table S7). The TPMs of CK09SX_02175 were 593.08 and 968.66 at 24 and 72 h culture, while the TPMs of the rest of the genes were lower than 20.

### 3.5. Motility

In *L. capsici* CK09, nineteen genes were homologous to the type four pili (T4P) biosynthesis genes in *L. enzymogenes* OH11 on the protein level (Table S8) [49]. In *L. capsici* CK09, the proteins encoded by CK09SX_05625 (PilC, platform protein), CK09SX_20190 (PilQ, outer membrane pore), CK09SX_20195 (PilP, mid periplasmic ring), CK09SX_20200 (PilO, lower periplasmic ring), CK09SX_20205 (PilN, lower periplasmic ring) and CK09SX_20210 (PilM, cytoplasmic ring) formed the basal body. The ATPase PilB (CK09SX_05630) was involved in pilus elongation while PilT (CK09SX_19075) and PilU (CK09SX_19070, CK09SX_05010) were essential for pilus retraction.

Moreover, the proteins encoded by CK09SX_05665, CK09SX_05670 and CK09SX_05675 carried a pilin domain (PF00114); thus, these genes were assigned as pilA. Several minor pilins, i.e., PilW (CK09SX_14390), PilE (CK09SX_17545), PilX (CK09SX_17555), PilW (CK09SX_17560), PilV (CK09SX_17565) and FimT (CK09SX_17570) were also identified. PilY1, encoded by CK09SX_17550, was found to be located at the tip of the pilus fiber [50] and was involved in surface adherence [51]. The minor pilins and PilY1 play critical roles in priming T4P assembly [52], whereas FimT mediates DNA uptake during bacterial natural transformation [53]. Thus, all the genes necessary for T4P biosynthesis were included in *L. capsici* CK09. The expression of all these genes was observed at both 24 and 72 h. The highest expression level was observed for CK09SX_05025 at 72 h.

### 3.6. Polysaccharide Biosynthesis

A putative poly-β-1,6-N-acetyl-D-glucosamine (PGA) biosynthesis gene cluster was also identified in the genome of *L. capsici* CK09 (Table S9). CK09SX_09935 showed 57.73% and 57.52% similarity to the PGA synthase in *Pectobacterium atrosepticum* [54] and *Klebsiella pneumoniae* [55], respectively. CK09SX_09940 showed 40.13% and 41.99% similarity to the PGA N-deacetylase in *P. atrosepticum* and *K. pneumoniae* on the protein level. On the other hand, CK09SX_09945 showed 26.96% and 4.84% similarity to the PGA transporter in *P. atrosepticum* and *K. pneumoniae*, whereas CK09SX_09930 was not homologous to the certain genes in *P. atrosepticum* and *K. pneumoniae*. However, the former contained a Porin_4 domain (PF13609) and the latter had a PgaD domain (PF13994). Thus, this gene cluster might be responsible for PGA synthesis and secretion. All the PGA biosynthesis genes showed insignificantly lower expression levels at 72 h compared to that at 24 h.

### 3.7. Secretion Systems

In Gram-negative bacteria, secretion systems can transport proteins across the cytoplasmic membrane to the periplasm or directly into the cytoplasm of host cells [56]. The genome of *L. capsici* CK09 carried the genes related to type two secretion system (T2SS), type three secretion system (T3SS), type four secretion system (T4SS), as well as type six secretion system (T6SS). Additionally, all the genes related to the secretion systems were expressed in broth at both 24 h and 72 h.

A gene cluster consisting of eleven genes was involved in T2SS synthesis (Table S10). The secretion channel at the outer membrane was composed of secretin GspD (CK09SX_05350). The assembly platform at the inner membrane consisted of GspF (CK09SX_05305), GspL (CK09SX_05335) and GspM (CK09SX_05340). The cytoplasmic ATPase (GspE, CK09SX_05300) was also found in CK09. GspG (CK09SX_05310) acted as the major pseudopilin, while GspH (CK09SX_05315), GspI (CK09SX_05320), GspJ (CK09SX_05325) and GspK (CK09SX_05330) formed the minor pseudopilins. The TPM of these genes were between 6.14 and 120.86. Among them, CK09SX_05320 showed a significantly higher expression level at 72 h culture, while no significant differences were observed in the expression levels of the rest of the genes.

*L. capsici* CK09 also contained a set of genes related to T3SS biosynthesis (Table S11). In CK09, SctD (CK09SX_23770), SctJ (CK09SX_23755) and SctI (CK09SX_23760) formed the basal body, while SctF (CK09SX_23720) was composed of the needle. SctU (CK09SX_23815), SctV (CK09SX_23775), SctR (CK09SX_23800), SctS (CK09SX_23805) and SctT (CK09SX_23810) formed the export apparatus. SctQ (CK09SX_23795) was located in the cytoplasm and linked to SctK and SctL. SctN (CK09SX_23740), SctL (CK09SX_23745), SctO (CK09SX_23735) and SctK (CK09SX_23750) formed the ATPase complex in the cytoplasm. Additionally, HpaP (CK09SX_23730), a T3SS substrate specificity switch, was found in CK09. Interestingly, a two-component system (TCS), including a histidine kinase (CK09SX_23785) and a response regulator (CK09SX_23790), was annotated in the T3SS biosynthesis cluster. However, the function of this TCS has not yet been elucidated. T3SS related genes were well-expressed at both 24 h and 72 h culture.

In *L. capsici* CK09, thirteen genes were identical to the T4SS biosynthesis genes in *L. enzymogenes* OH11 [57] at protein level (Table S12). With this system, VirB3 (CK09SX_13930), VirB6 (CK09SX_13910) and VirB8 (CK09SX_13960) formed the inner membrane complex (IMC). The IMC connects the ATPase energy center in cytoplasm and the outer membrane core complex. The former consisted of VirD4 (CK09SX_11415), VirB4 (CK09SX_13925) and VirB11 (CK09SX_13945), whereas the latter included VirB7 (CK09SX_13965), VirB9 (CK09SX_13955) and VirB10 (CK09SX_13950). VirB2 (CK09SX_13935) generated the pilin, while VirB5 (CK09SX_13915, CK09SX_13920) was localized at the tip of the pilus. VirB1 (CK09SX_13940) was identified as a periplasmic protein, but its function remains to be determined. Besides CK09SX_13955 and CK09SX_13960, which showed lower expression levels at 72 h culture, the expression levels of these genes were similar at both sampling times.

T6SS was identified in *L. enzymogenes* OH11 [58]. The corresponding cluster in *L. capsici* CK09 was composed of 14 genes (Table S13). On the protein level, TssE (CK09SX_12655), TssF (CK09SX_12650), TssG (CK09SX_12645) and TssK (CK09SX_12685) formed a baseplate at the inner membrane. The tail tube/sheath complex, which was built on the baseplate through the inner membrane, was composed of the tube protein Hcp (CK09SX_12660), the sheath TssB (CK09SX_12670) and TssC (CK09SX_12665). The protein encoded by CK09SX_12735 contained an ImpA_N domain (PF06812) at the N-terminal. Thus, it was identified as a tube cap protein TssA. On the other side, TssJ (CK09SX_12680), TssL (CK09SX_12690) and TssM (CK09SX_12775) formed a membrane complex, which was anchored on the baseplate, spanning the periplasm, and reaching the outer membrane. Additionally, VgrG (CK09SX_12635) was located on the top of the tube and formed a spike. The protein encoded by CK09SX_12675 contained a signal for secretion (SignalIP) and was predicted to be a lipoprotein. However, its function is still unknown. Most T6SS genes were expressed constitutively with one exception, CK09SX_12660.

### 3.8. Secretome

Bacterial cells can export a variety of proteins across the cell membrane. In *L. capsici* CK09, 840 proteins, constituting 16.8% of the genome, were predicted to harbor signal peptides for secretion (Table S14). Among them, 805 (95.83%) proteins, including 226 lipoproteins and 6 pilin-like peptides, were secreted by the Sec system; 35 proteins, including 3 lipoproteins, were secreted via the twin arginine translocation (Tat). The transcriptomic data validated the expression of 832 out of these 840 genes.

The pfam annotation results indicated the secretome protein function mainly classified in beta-lactamase (PF00144), peptidase family M23 (PF01551) and WD40-like beta propeller repeat (PF07676). The former is involved in beta-lactams inactivation [59], the middle is associated with protein degradation, and the latter is abundant in eukaryotes and has diverse fundamental functions [60], while its function in prokaryotes is still uncharacterized. Other peptidases (PF00082, PF01435, PF03572, PF04389, PF05649, PF07504, PF07687, PF10459, PF12146, PF13365 and PF13688), alpha-lytic protease (PF02983), oligopeptidase (PF00326) and dipeptidase (PF00930, PF01244) were also identified in the secretome. Additionally, alpha/beta hydrolase (PF00561, PF12697)-, cellulase (PF00150)- and mannosidase (PF07971)-related genes were predicted to be secreted. Signal peptides were also found in nuclease (PF03372), amidohydrolase (PF01979) and esterase (PF10503).

## 4. Discussion

In recent years, an increasing number of studies have focused on the antimicrobial activities of *L. capsici* [21,27,61]. However, only one genome was analyzed in depth with an emphasis on antifungal properties and stress responses [62]. In this paper, we aimed to investigate not only the antimicrobial potential, but also the soil adaption of *L. capsici* CK09.

Advancements in cost-effective, high-throughput DNA sequencing technologies have enabled genomic comparison between bacterial strains based on whole-genome sequencing. Since then, ANI analysis has been widely utilized as a reliable tool for taxonomic reclassification [63,64]. Recently, ANI analysis has been applied in the identification of novel species in *Lysobacter* [65,66]. In a previous study, *L. capsici* CK09 was recognized based on the 16S rRNA gene [35]. In this study, the ANI value confirmed the genetic relationship between *L. capsici* CK09 and various *L. capsici* strains. This ANI analysis also suggested the reclassification of several strains. As it provides a higher resolution than the 16S rRNA gene on the species level since the latter is highly conserved [67], the taxonomic reclassification of the *Lysobacter* genus should be investigated further in the future.

### 4.1. Biocontrol Potential

*Lysobacter* is attracting increasing attention for its ability to produce diverse bioactive nature products [68]. The fungal cell wall, which determines the shape and strength of the fungus, is composed of polysaccharides and proteins and is essential for fungal viability [69]. The polysaccharides, including chitin and β-1,3-glucan, form the inner wall skeleton, which provide the mechanical strength of the cell wall [69]. The production of chitinase and glucanase, which break down chitin and glucan, respectively, by *L. capsici* CK09 contribute to its biocontrol activity against the fungal phytopathogen [70,71,72]. HSAF, a broad-spectrum antifungal activity produced by *L. capsici* CK09, can interfere in ceramide synthesis and polarized hyphal growth [73], inhibit conidia germination [74] and trigger autophagy-mediated degradation [75]. Thus, the production of HSAF, together with chitinases and β-1,3-glucanases, exert the contact-independent antifungal activity of *L. capsici* CK09. The expression of antifungal genes in the absence of phytopathogen indicates that the induction of the fungus is not required.

Several contact-dependent antimicrobial mechanisms, such as T4SS and T6SS, play roles in *L. capsici* CK09 antibacterial activities through the direct injection of toxic proteins and competition for metal ions. T4SS facilitate the transport of DNA and proteins either directly into the environment or into target cells [76]. T4SS-mediated horizontal DNA transfer, also known as conjugation, spreads mobile genetic elements between bacteria, and is a key process for bacterial evolution and for antibiotic-resistant gene transfer [77]. Another important function of T4SS is the injection of antibacterial proteins directly into neighboring cells. For example, *Xanthomonas citri* kills *E. coli* cells in a T4SS-dependent manner in various nutrient conditions [78], while in *L. enzymogenes*, T4SS supports its bactericidal activity against *Pseudomonas fluorescens* and *Pseudomonas protegens* [57]. T6SS is also a well-known contact-dependent antimicrobial apparatus. The effectors produced by T6SS act as peptidoglycan hydrolase, (phosphor-)lipase or nuclease and then inhibit the growth of competitors [79]. T6SS also works as a channel to compete with metal ions in the milieu, such as zinc, manganese, iron and copper [80]. Thus, T4SS and T6SS contribute to the antibacterial activities of *L. capsici* CK09 via bactericidal action and/or nutrient competition.

### 4.2. Adaption to Soil

Soil nutrients, such as carbon and nitrogen sources, are positively correlated to bacterial survival [81]. *L. capsici* CK09 is able to utilize a variety of carbon sources, such as N-acetyl-D-glucosamine and glucose, according to the data from BIOLOG GEN III [35]. N-acetyl-D-glucosamine is the product of chitin degradation, while glucose is the building block of β-1,3-glucan. Thus, the production of chitinase and glucanase provide *L. capsici* CK09 with not only biocontrol activity, but also available nutrients. The presence of various carbohydrate-active enzymes in the soil-borne bacteria *L. capsici* CK09 also suggests the role of nutrient acquisition in soil adaption. The primary role of glycoside hydrolases is the hydrolytic breakdown of carbohydrates [82], subsequently providing an accessible carbon source for microbial growth. Thus, the presence and expression of chitinase and β-1,3-glucanase (Table S1), along with other glycoside hydrolases (Table S2), can improve the nutrient availability of *L. capsici* CK09 in soil.

Heterotroph bacteria depend on nutrients in the milieu for their survival and growth. Although cellulose, chitin, protein and nucleic acid are abundant biopolymers in the environment, they are not able to be taken up through the bacterial membrane, since the transporters only permit the import of compounds less than 6 KDa [83]. Thus, the export of degrading enzymes into the extracellular environment is required for the utilization of these high-molecular compounds. Diverse enzymes, such as glycoside hydrolases, protease/peptidase and nuclease, are found to harbor signal peptides, which could be recognized and exported by the Sec translocon or the Tat system. Sec and Tat are two universal protein secretory systems that are essential for protein export across the plasma membrane [84,85]. The proteins transported by the Sec system are in the unfolded states [84], whereas the Tat system secretes folded proteins [85]. T2SS can also mediate the translocation of periplasm proteins through the outer membrane [86]. It has been reported that T2SS supports the secretion of degrading enzymes, such as amidase, endoglucanase, chitinase, peptidase, lipase and phosphatase [87], and promotes bacterial nutrient acquisition. Thus, the secretion of lytic enzymes through these secretion systems provides a competitive advantage of enabling *L. capsici* CK09 to survive in soil.

Extensive surfaces, such as soil particles, root surfaces, fungal hyphae and microplastics, dominate in agriculture soil [88,89,90]. The colonization and biofilm formation of bacterial cells on surfaces in soil allow them to utilize higher concentrations of nutrients, to develop a greater diversity and evenness in the microbial community, to induce elevated metabolic activity and to protect bacterial cells from stresses such as toxic compounds [88,91]. In bacteria, type IV pili (T4P) are involved in twitching motility and solid surface sensing [92]. Bacterial cells tend to be motile under nutrient-depleted conditions and to be sessile in nutrient-rich environments [93]. Thus, for the biocontrol strain *L. capsici* CK09, T4P-mediated dispersal is needed for their exploration of nutrients in soil, for reaching crop roots and for microcolony formation on the root surfaces. The root surface is a nutrient-rich hotspot due to the release of multiple metabolites by plants, such as carbohydrates, amino acids and organic acids [94]. Then, the biofilm is developed with the help of T3SS and PGA. A three-step model of biofilm formation, including aggregation and attachment, growth and accumulation, and disaggregation and detachment, was recently proposed [91]. T3SS is widely utilized by pathogenic bacteria to inject virulence factors directly into host cells [95] and is also involved in the initial attachment of bacteria to host cells during the first stage of contact [96,97]. In the next stage, the biosynthesis of polysaccharide facilitates robust biofilm formation, thus protecting bacterial cells from hostile environments [98]. PGA is a polysaccharide produced by various bacteria and was found to be involved in biofilm formation and antibiotic resistance [54,99]. Thus, T4P-mediated motility, T3SS-mediated initial adherence and PGA-mediated biofilm formation on the surfaces of plant roots and fungal hyphae provide a selective advantage enabling *L. capsici* CK09 to live in soil.

In conclusion, *L. capsici* CK09 possesses various biocontrol traits, such as lytic enzymes, HSAF biosynthesis, T4SS and T6SS. Additionally, the secretion of degrading enzymes and the formation of biofilm contribute to *L. capsici* CK09’s adaption in soil. By analyzing the genome of *L. capsici* CK09, this study provides a mechanistic understanding of the application of this eco-friendly biocontrol strain in protecting crops against fungal pathogens, enabling a further reduction in the use of toxic fungicides in agriculture. Future work should be conduct to elucidate the influence of soil properties, i.e., biotic and abiotic factors, on the survival of *L. capsici* CK09 in soil ecosystem, and to develop commercial biocontrol agent using biopolymeric materials as carriers.

## Data Availability

The genomic information and transcriptomic sequencing data were submitted to the National Genomic Data Center with the accession numbers GWHCBIO01000000 and CRA010911.

## Supplementary Materials

The following supporting information can be downloaded at: https://www.mdpi.com/article/10.3390/microorganisms11071768/s1, Table S1: Chitinase- and β-glucanase-related genes in *L. capsici* CK09, Table S2: CAZyme annotation and expression pattern of carbohydrate-active enzymes in *L. capsici* CK09, Table S3: Secondary metabolite biosynthesis gene clusters in *L. capsici* CK09 identified via antiSMASH, Table S4: Yellow pigment biosynthesis gene cluster in *L. capsici* CK09, Table S5: WAP-8294A2 biosynthesis gene cluster in *L. capsici* CK09, Table S6: HSAF biosynthesis gene cluster in *L. capsici* CK09, Table S7: NRPS genes identified in *L. capsici* CK09, Table S8: Type 4 pili biosynthesis genes in *L. capsici* CK09, Table S9: PGA biosynthesis genes in *L. capsici* CK09, Table S10: T2SS biosynthesis genes in *L. capsici* CK09, Table S11: T3SS biosynthesis genes in *L. capsici,* CK09, Table S12: T4SS biosynthesis genes in *L. capsici* CK09, Table S13: T6SS biosynthesis genes in *L. capsici* CK09, Table S14: Predicted secretome in *L. capsici* CK09.
